# Beyond a deficit-based approach: Characterizing typologies of assets for cisgender and transgender female sex workers and their relationship with syndemic health outcomes

**DOI:** 10.1371/journal.pgph.0002314

**Published:** 2023-08-30

**Authors:** Beth J. Maclin, Yan Wang, Carlos Rodriguez-Diaz, Yeycy Donastorg, Martha Perez, Hoisex Gomez, Clare Barrington, Deanna Kerrigan

**Affiliations:** 1 Department of Prevention and Community Health, Milken Institute School of Public Health, The George Washington University, Washington, District of Columbia, United States of America; 2 Behavioral & Community Health Sciences, School of Public Health, University of Pittsburgh, Pittsburgh, Pennsylvania, United States of America; 3 Instituto Dermatológico y Cirugía de la Piel, HIV Vaccine Trials Research Unit, Santo Domingo, Dominican Republic; 4 Department of Health Behavior, Gillings School of Global Public Health, University of North Carolina, Chapel Hill, North Carolina, United States of America; University of Toronto Temerty Faculty of Medicine, CANADA

## Abstract

Female sex workers (FSWs) live and work at the intersection of multiple marginalized identities that place them at greater risk for various negative health outcomes. Resilience theory asserts that an individual or community needs assets from which they can draw in response to stressors, such as chronic discrimination and abuse. This study characterizes and compares patterns of assets among cisgender and transgender FSWs living with HIV in the Dominican Republic and their relations with syndemic health outcomes. With Latent Class Analysis, we used companion cross-sectional datasets comprised of cisgender and transgender FSWs (N = 211 and 100, respectively) to estimate typologies of interpersonal, community, and institutional assets. We used multivariate logistic regression to model the relationship between class membership and HIV care and treatment, mental health, violence exposure, and substance use outcomes, respectively. Among cisgender FSWs, we identified three classes: Internal and External Multilevel Assets (Class 1); External Institutional Assets (Class 2); and Low Reported Assets (Class 3). Compared to Class 3, Class 1 membership among cisgender FSWs was significantly associated with ART adherence and marginally associated with viral suppression, and Class 2 membership was marginally associated with currently taking ART. We identified two classes in the transgender sample: Internal and External Multilevel Assets (Class 1) and External Institutional Assets (Class 2). Class 1 membership among transgender FSWs was significantly associated with ART adherence and marginally associated with current ART use and physical or sexual violence, compared to Class 2. Having a variety of assets may explain the ability of some FSWs to more effectively engage with healthcare and maintain their HIV medication regimen. Future interventions should seek to expand FSWs’ interpersonal and community assets, both from within and outside of the sex worker community, to bolster their ability to care for themselves and their community.

## Introduction

Cisgender and transgender female sex workers (FSWs) face syndemics of HIV, mental illness, substance use, and violence [1–9] as a result of societal marginalization driven by sexism, sex work stigma, HIV stigma, and transphobia [1,10–13]. It is important to understand sources of vulnerability and the resulting impact on health outcomes for this group. However, deficit-based approaches focusing on risk factors alone largely ignore the agency and power of FSWs as individuals and communities. An asset-based approach, in contrast, reorients the lens through which we understand this group’s experiences and resources in order to identify protective factors that may promote overall health and wellbeing.

Resilience theory presents a foundation on which to employ an asset-based analysis. While there is not a single agreed-upon definition of resilience, it is generally understood to be a process to achieve better-than-expected outcomes and/or sustain wellbeing in the face of acute or chronic adversity [14–16]. The Resilience Activation Framework (RAF) acknowledges how the process of resilience occurs at the micro, macro, and meso levels and is dependent on an individual’s or community’s assets [17]. Assets can include biopsychosocial, economic, political, cultural, and structural or institutional resources [14,17]. Another way to think about assets is through the lens of social capital, which suggests that assets have both relational (social) and material (resources) characteristics and benefits [18].

The RAF also considers the source of assets, including from the individual, community, or beyond [17]. Resilience assets interact within and across levels and domains [19], which is critical because ample evidence points to the importance of social connections (family, friends, coworkers, classmates, etc.) when facing adversity and how these relationships are the central point for the mediating processes of resilience [16,20–22]. The study of social capital separates these connections into two categories: bonding and bridging. *Bonding* occurs within a closed network of homogeneous people, while *bridging* involves heterogeneous groups of people within an open network [23–25]. Further, when considering social ecologies of resilience, the interconnectedness between the personal and environmental factors, i.e., social structures or services, is the most central and powerful resilience resource in the mediating processes [16,26,27].

Some studies have examined a variety of assets, including social and economic, held by FSWs and how FSWs employ them to mitigate the chronic adversity they face [28–34]. In India, for example, Ganju and Saggurti [28] identified how transgender FSWs used social assets to respond to violence they face and to address poor mental health. Through their peer networks and collective action, transgender FSWs were able to confront clients and police who harassed and abused them as well as gained support in terms of self-acceptance and care for mental and physical health [28]. Similarly, cisgender FSWs in Tanzania who work in bars and participated in the Project Shikamana intervention developed strategies using social and economic assets to protect each other and reduce harm from clients [29]. Participants mobilized their collective agency and social assets to address or subvert threats of violence associated with alcohol consumption throughout the sex exchange process. While attracting clients at a bar, participants described working with the barmaids to have them secretly replace the beer in bottles bought by potential clients with water to remain sober while working. During the negotiation process, participants described how they would pool money together if a sex worker was unable to pay back a refused client for the drinks he purchased in order to reduce the likelihood of violence [29].

In the Dominican Republic (DR), marginalized communities have been organizing and advocating for their rights and improved living and working conditions through organizations like Movimiento de Mujeres Unidas (MODEMU), a civil society organization of current and former FSWs founded in 1996, and Trans Siempre Amigas (TRANSSA), an organization focused on promoting and protecting the rights of trans people in the DR since 2006 [35,36]. There are an estimated 87,000–98,000 cisgender FSWs and 3,900–9,000 transgender women, many of whom engage in sex work, in the DR [37,38]. [Cisgender] FSWs have an estimated HIV prevalence of three percent [38], and transgender women, many of whom reported engaging in sex work, have an estimated prevalence rate of 35.8% [39]; in contrast, the national HIV prevalence rate is 0.9 for adults aged 15–49 years old [40]. While sex work is not illegal in the DR, the combination of sex work stigma, sexism, transphobia, and HIV stigma create an environment where FSWs living with HIV face discrimination that impacts their access to social, economic, legal, and health services [37,41–43].

The chronic adversity cisgender and transgender FSWs living with HIV experience necessitates a level of resilience to endure the resulting syndemics. Based on resilience theory, this process requires assets from which FSWs can draw [16,17,19,44]. This is the first study we know of that characterizes and compares typologies of assets among cisgender and transgender FSWs living with HIV and examines how these typologies are associated with syndemic health outcomes. We hypothesized that holding social and/or economic assets will be protective for FSWs against syndemic health outcomes. Further, we hypothesized that the cisgender and transgender samples will produce different typologies, with the cisgender-specific typology reflecting more asset ownership.

## Materials and methods

### Study design & sample

This is a cross-sectional study. We used a combined dataset from two companion studies with cisgender and transgender FSWs living with HIV in Santo Domingo, DR (N = 211 and 100, respectively). The parent study with cisgender FSWs living with HIV included three waves of data collection; we used data from wave II for this analysis, which was collected in December 2018-November 2019. Data for the supplement study with transgender FSWs were collected in January-September 2019.

Recruitment details for both cisgender participants [33,45,46] and transgender participants [34,47] are published elsewhere. For both samples, data collection occurred at the Instituto Dermatológico y Cirugía de la Piel (IDCP) in Santo Domingo, DR. Participants completed a survey including demographic, behavioral, health, and socio-structural questions, and provided blood for viral load testing.

Both studies were reviewed and approved by the Johns Hopkins University (JHU) Bloomberg School of Public Health Institutional Review Board (IRB) and the IDCP. The George Washington University IRB deferred to the JHU IRB. All participants were 18 years or older, verbally consented to participate, and were compensated for their time with $10 USD (paid in DR pesos).

### Measures

#### Asset items

In this study, assets are defined as concrete preconditions (material, social, psychological, organizational, and/or institutional resources) that improve access to opportunities and enable individuals and communities to take advantage of those opportunities if they wish to do so [48–52]. Seven categorical and continuous variables representing social and economic assets that span the RAF’s interpersonal, community, and institutional levels were included; these measures can also be viewed as coming from within or outside of the sex worker community.

*Interpersonal*: 1) Intervention peer interaction: In the past 6 months, have you had contact with a peer educator/navigator about HIV/AIDS? (yes versus no); 2) Cash transfers or remittances: receipt of cash transfer of any kind from another individual (yes versus no).

*Community*: 1) Social cohesion: a variable based on an 11-item scale assessing relationships of trust, mutual aid, and support specific to sex worker communities. A sum score was created, ranging within 11–44. This scale had a Cronbach’s alpha of 0.84 for the DR [53]; 2) Sex worker group participation: In the past six months, how often have you participated in an organized group with other sex workers? (ever versus never); 3) Other community group membership: a variable based on a four-question index regarding participation (none, member, or leader) in religious groups, clubs, cultural activities, and neighborhood associations. A sum score was created, ranging from 4–12; 4) HIV support group: In the past six months, have you participated in a support group with other people living with HIV? (yes versus no).

*Institutional*: 1) A four-question index of institutional resource ownership. including voter registration card, bank account, health insurance, and birth certificate. A sum index score was created (range 4–8).

#### Syndemic health outcomes

*HIV continuum of care*: 1) Viral suppression (<400 copies/mL versus ≥400 copies/mL); 2) Current ART use (yes versus no); 3) ART adherence over the past four days (perfect adherence, e.g. taking all ART medicines as prescribed, versus not); 4) ART disruption in the past six months (ever stopped taking ART versus never).

*Mental Health*: 1) Anxiety: Normal (score of 0–7) versus Borderline Abnormal or Abnormal (score of ≥8) based on the anxiety-specific module from the Hospital Anxiety and Depression Scale (HADS-A) [54]; 2) Depression: Minimal or Mild Depression (score of ≤9) versus Moderate to Severe Depression (score of ≥10) based on the Patient Health Questionnaire-9 (PHQ-9) [55].

*Violence Exposure*: 1) Physical/Sexual: Any physical and/or sexual violence from a regular partner, client, or police in the past six months (yes versus no/unknown) [56–58]. Only participants who reported having a regular partner, new client, or regular client in the past 30 days were asked the follow-up questions regarding physical and sexual violence in the past six months from the respective perpetrators. If participants answered no to the initial question of having a regular partner, new client, or regular client, they were marked as no/unknown for the follow-up questions on physical and sexual violence exposure.

*Substance Use*: 1) Alcohol Use Disorder (AUD) [59]: "Not at risk for AUD" (score 0–2) versus "At risk for AUD" (score 3–15), based on three questions comprising a 15-point scale about the frequency of drinking alcohol per week, the number of drinks consumed per event, and the frequency of consuming six or more drinks at a time [60]; 2) Illicit drug use: Any use versus none of marijuana, crack, cocaine, heroin, ecstasy, and/or other drugs in the past six months.

#### Participants characteristics

*Sociodemographic*: 1) Age; 2) Relationship status (partnered [living with partner or married] versus not [divorced, separated, widowed, single/never married]); 3) Educational attainment based on the last grade completed by school level; 4) Average monthly income in the past six months from all revenue sources; and 5) Birthplace (Santo Domingo, DR, versus elsewhere).

*Occupational Characteristics*: 1) Primary occupation (sex worker versus other); 2) Number of years exchanging sex for money; 3) Sex work employment status (self-employed versus employed part- or full-time); and 4) Traveling to exchange sex for money outside of one’s city in the past six months (yes versus no).

### Analysis

We conducted chi-square statistical tests and t-tests to compare the cisgender and transgender samples on the asset, health outcome, sociodemographic, and occupational variables. We also conducted chi-square statistical tests and t-tests to compare the classes within each sample on sociodemographic and occupational variables in order to identify covariates for the logistic regressions.

We conducted Latent Class Analysis (LCA) to identify patterns of assets for cisgender and transgender FSWs. The following criteria were used to select the number of classes: Akaike Information Criterion (AIC), Bayesian Information Criterion (BIC), and sample size-adjusted BIC (aBIC), of which smaller values are better; the Vuong-Lo-Mendell-Rubin Likelihood Ratio Test (VLMR-LRT) and Bootstrapped Likelihood Ratio Test (BLRT), where a significant p-value suggests that m classes fits the data significantly better than m-1 classes; entropy values greater than 0.7; and the conceptual interpretation and usefulness of the latent classes [61–63]. To ensure model identification, models were run with 2,000 random starting values. The analyses were conducted for each group (cisgender and transgender), separately, given that configural invariance (e.g. number of latent classes) for the LCAs does not hold across the cisgender and transgender groups.

We conducted multivariate logistic regression or multinomial logistic regression to assess the association between latent class membership and each syndemic health outcome separately for each group, controlling for relevant covariates. For parsimonious purposes, we only controlled for covariates with significance of p<0.05 in the chi-square statistical tests and t-tests. Further, we conducted post-hoc regression analyses for individual health outcome results to examine potential syndemic-related explanations.

We used Stata version 16.1 [64] and Mplus (Version 8.7-Linux) [65]. Statistical significance was defined as p<0.05; marginal significance was defined as p<0.10.

## Results

### Group characteristics

**Table 1** presents characteristics and syndemic health outcomes for both samples. The transgender participants completed more education (i.e. 74.00% completed at least one grade level in secondary school or higher vs. 38.86%, p<0.001), reported higher average monthly income (mean 18,730 Dominican pesos ($340 USD) vs. 9,640 pesos ($175 USD), p<0.001), and more often reported being born in Santo Domingo (72.00% vs. 59.7%, p<0.05). In addition, a larger proportion of cisgender participants were currently partnered (41.23% vs. 16.00%, p<0.001), and they were older than their transgender counterparts (mean 40.91 years vs. 34.08 years, p<0.001).

**Table 1:** Distribution of sample characteristics and syndemic health outcomes for cisgender and transgender samples (mean (SD) or frequency (%)).

	Transgender (N = 100)	Cisgender (N = 211)
Socio-Demographic		
Age (years)***	34.08 (9.96)	40.91 (8.93)
Relationship status***		
Currently partnered	16 (16%)	87 (41.23%)
Not partnered	84 (84%)	124 (58.77%)
Last school grade completed***		
None	1 (1%)	9 (4.27%)
Primary	25 (25%)	120 (56.87%)
Secondary	52 (52%)	71 (33.65%)
Post-secondary	22 (22%)	11 (5.21%)
Average monthly income from all income sources in past six months (DR pesos/1000)***	18.73 (17.01)	9.64 (6.64)
Born in Santo Domingo, DR*		
Yes	72 (72%)	126 (59.7%)
No	28 (28%)	85 (40.28%)
Occupation Characteristics		
Primary occupation		
Sex Worker	85 (85%)	184 (87.20%)
Other	15 (15%)	27 (12.80%)
Number of years as a sex worker**	16.47 (9.69)	19.56 (9.30)
Sex Work Employment Status		
Self-employed	87 (87%)	196 (92.89%)
Employed full- or part-time	13 (13%)	15 (7.11%)
In the past 6 months, have you traveled outside of the city where you reside specifically to exchange sex for money elsewhere?*		
Yes	26 (26%)	32 (15.17%)
No	74 (74%)	176 (83.41%)
Syndemic Health Outcomes		
Viral suppression (<400 copies/mL)*		
Yes	64 (64%)	160 (75.83%)
No	36 (36%)	51 (24.17%)
Currently taking ART***		
Yes	84 (84%)	203 (96.21%)
No/Missing	16 (16%)	8 (3.79%)
ART adherence in past 4 days		
Perfect adherence	44 (44%)	84 (39.81%)
Not perfect adherence	56 (56%)	127 (60.19%)
Ever stopped taking ART in past 6 months*		
Yes	13 (13%)	54 (25.59%)
No/Missing	87 (87%)	157 (74.41%)
HADS-A		
Borderline Abnormal or Abnormal (≥8)	34 (34%)	85 (40.28%)
Normal (≤7)	66 (66%)	126 (59.72%)
PHQ-9		
Moderate to Severe Depression (≥10)	24 (24%)	54 (25.59%)
Minimal to Mild Depression (≤9)	76 (76%)	157 (74.41%)
Physical and/or sexual violence in past 6 months***		
Any	36 (36%)	24 (11.37%)
None/Missing	64 (64%)	187 (88.63%)
Alcohol Use Disorder (AUD) risk		
At risk of Alcohol Use Disorder	77 (77%)	140 (66.35%)
Not at risk	23 (23%)	71 (33.65%)
Illicit drug use in past 6 months***		
Any	55 (55%)	27 (12.80%)
None	45 (45%)	184 (87.20%)

* = p<0.05

**p<0.01

*** = p<0.001.

For occupation characteristics, more transgender participants said they traveled to exchange sex for money (26.00% vs. 15.17%, p<0.05), and cisgender participants engaged in sex work for more years (mean 19.56 vs. 16.47, p<0.01).

A significantly larger proportion of cisgender participants were currently taking ART (96.21% vs. 84.00%, p<0.001), reported ART disruption in the past six months (25.59% vs. 13%, p<0.05), and were virally suppressed (75.83% vs. 64.00%, p<0.05). In contrast, more transgender participants reported any physical or sexual violence in the past six months (36.00% versus 11.37%, p<0.001) and any illicit drug use in the past six months (55.00% vs. 12.80%, p<0.001).

**Table 2** summarizes the asset items for both groups. A larger proportion of transgender participants reported participating in an organized sex worker group (51.00% versus 33.18%, p<0.01) and receipt of cash transfers (52.00% versus 30.33%, p<0.001) than their cisgender counterparts.

**Table 2:** Distribution & probability for each asset item and proportion of respondents by class for cisgender and transgender samples.

	Transgender Sample (N=100)			Cisgender Sample (N=211)			
	Overall Sample	External Institutional Assets	Internal & External Multilevel Assets	Overall Sample	Low Reported Assets	External Institutional Assets	Internal & External Multilevel Assets
**Proportion of Sample**		0.5738	0.4262		0.0921	0.5415	0.3664
**Continuous Items**	mean (SD)			mean (SD)			
Social cohesion scale (11-44)	27.32 (4.02)	27.04	27.70	27.49 (4.25)	25.95	25.80	30.38
Group membership index (4-12)	4.66 (0.99)	4.47	4.91	4.90 (1.01)	5.00	4.61	5.30
Institutional assets index (4-8)	7.19 (0.91)	7.33	7.00	7.05 (1.02)	4.46	7.21	7.48
**Categorical Items**	n (%)			n (%)			
Intervention peer interaction							
Yes	37 (37%)			82 (38.86%)			
No	63 (63%)	0.19	**0.61**	129 (61.14%)	0.37	0.28	**0.55**
Attended HIV support group							
Yes	43 (43%)			87 (41.23%)			
No	57 (57%)	0.01	**1.00**	124 (58.77%)	0.16	0.19	**0.81**
SW group participation**							
Any	51 (51%)			70 (33.18%)			
None	49 (49%)	0.35	**0.72**	141 (66.82%)	0.16	0.11	**0.70**
Received cash transfer***							
Yes	52 (52%)			64 (30.33%)			
No	48 (48%)	0.46	**0.61**	147 (69.67%)	0.05	0.32	0.35

**Items in bold** note when members of this class have greater than 0.5 probability of reporting this (categorical) asset.

* = p<0.05

**p<0.01

***p<0.001, when comparing each variable overall between the cisgender and transgender samples.

### Latent class analysis

#### Transgender sample

A summary of the model fitness criteria for the transgender-specific models is presented in **Table 3**. We chose the two-class model because the three-class model had a non-significant VLMR-LRT, and the smallest class comprised only one percent of the participants. The two classes are identified as External Institutional Assets (57.38% of participants) and Internal and External Multilevel Assets (42.62%). **Table 2** presents the distributions for each item in each class. The External Institutional Assets class shows participants assigned to this class likely have an above-average score for the institutional assets index item (7.33 versus 7.19); this is the distinguishing feature of this class, which supports the class name as the source is external to the sex worker community and sits at the institutional level. The Internal and External Multilevel Assets class also has a relatively high average score for the institutional assets index item (7.00 out of 8). The items that distinguish it from the other class are intervention peer interaction, HIV support group attendance, sex worker group participation, and receipt of cash transfers, all of which have probabilities greater than 0.50. The assets come from sources internal and external to the FSW community, as well as the interpersonal, group, and institutional levels.

**Table 3:** Comparisons of LCA model among transgender and cisgender samples, separately*.

	2-Class Model	3-Class Model	4-Class Model
Transgender Participants (N = 100)			
AIC	*1642*.*67*	1617.833	
BIC	*1689*.*563*	1685.567	
aBIC	*1632*.*714*	1603.453	
Vuong-Lo-Mendell-Rubin Likelihood Ratio Test	*0*.*0062*	0.1825	
Bootstrapped Likelihood Ratio Test	*<0*.*001*	<0.001	
Entropy	*0*.*973*	0.885	
Proportion for smallest class	*0*.*42616*	0.01	
Cisgender Participants (N = 211)			
AIC	3425.617	*3348*.*903*	3328.102
BIC	3485.951	*3436*.*052*	3442.065
aBIC	3428.916	*3353*.*668*	3334.332
Vuong-Lo-Mendell-Rubin Likelihood Ratio Test	0.0001	*0*.*0085*	0.0635
Bootstrapped Likelihood Ratio Test	<0.001	<0.001	<0.001
Entropy	0.973	*0*.*794*	0.795
Proportion for smallest class	0.09619	*0*.*09212*	0.09198

*Italics denote the model selected as best fitting the data.

**Table 4** presents the associations between class membership and syndemic health outcomes. Participants assigned to the Internal and External Multilevel Assets class had significantly greater odds of reporting perfect ART adherence over the past four days compared to the External Institutional Assets class (adjusted OR (aOR) = 2.91; 95%CI 1.25, 6.78; p<0.05), adjusting for covariates. Being in the Internal and External Multilevel Assets class marginally increased the odds of currently taking ART (aOR = 2.83; 95%CI 0.82, 9.73; p<0.10) and reporting any physical or sexual violence in the past six months (aOR = 2.11; 95%CI 0.90, 4.95; p<0.10), compared to the External Institutional Assets class.

**Table 4:** Distribution of syndemic health outcomes across classes (n(%)) and association between class membership and syndemic health outcomes for transgender sample with external institutional assets class as reference.

	**External Institutional Assets (n (%))**	**Internal & External Multilevel Assets (n (%))**	**Adjusted**^**a**^ **OR** (**95% CI)**	**p-value**
**Proportion of assigned participants**	**57 (57%)**	**43 (43%)**		
Viral suppression				
Yes	34 (59.65%)	30 (69.77%)		
No	23 (40.35%)	13 (30.23%)	1.77 (0.74, 4.23)	0.197
ART adherence in past 4 days				
Perfect adherence	19 (33.33%)	25 (58.14%)		
Not perfect adherence	38 (66.67%)	18 (41.86%)	2.91 (1.25, 6.78)	0.013**
Stopped taking ART in past 6 months				
Ever	6 (10.53%)	7 (16.28%)		
Never	51 (89.47%)	36 (83.72%)	1.60 (0.48, 5.31)	0.445
Currently taking ART				
Yes	45 (78.95%)	39 (90.70%)		
No	12 (21.05%)	4 (9.30%)	2.83 (0.82, 9.73)	0.099*
HADS-A				
Borderline Abnormal | Abnormal	15 (26.32%)	19 (44.19%)		
Normal	42 (73.68%)	24 (55.81%)	2.05 (0.87, 4.86)	0.103
PHQ-9				
Moderate to Severe depression	12 (21.05%)	12 (27.91%)		
Minimal to Mild depression	45 (78.95%)	31 (72.09%)	1.19 (0.46, 3.09)	0.715
Physical and/or sexual violence in past 6 months				
Any	16 (28.07%)	20 (46.51%)		
None	41 (71.93%)	23 (53.49%)	2.11 (0.90, 4.95)	0.086*
Alcohol Use Disorder (AUD) Risk				
At risk	45 (78.95%)	32 (74.42%)		
Not at risk	12 (21.05%)	11 (25.58%)	0.64 (0.24, 1.72)	0.381
Illicit drug use in past 6 months				
Any	28 (49.12%)	27 (62.79%)		
None	29 (50.88%)	16 (37.21%)	1.56 (0.68, 3.58)	0.293

* = p<0.10,

** = p<0.05,

*** = p<0.01.

a = Adjusted for birth place (Santo Domingo, DR, or elsewhere).

**Cisgender sample.** A summary of the model fitness criteria for models considered from the cisgender sample models is presented in **Table 3**. The three-class model fits the data best based on AIC, BIC, and aBIC as all three are smaller than compared to the two-class model. Further, the VLMR-LRT shows that the three-class model fits the data better than the two-class model, but the four-class model does not improve the model fit compared to the three-class model. When considering the classes for the three- and four-class models conceptually, the additional class in the four-class model did not provide new information compared to the three-class model. Rather, there would have been two classes named Internal and External Multilevel Assets, signifying comparable conceptual content. Thus, we selected the three-class model.

The three classes are: 1) Low Reported Assets (9.21% of participants), 2) External Institutional Assets (54.15%), and 3) Internal and External Multilevel Assets (36.64%). For the first class, none of the items are high: the social cohesion scale score is below average; both index scores are close to the low end of the possible range, and none of the binary variables have a probability of greater than 0.50. As with the transgender sample, the sole distinguishing item for the second class is the institutional assets index, which is above average (7.21 versus 7.05). Finally, for the third class, the social cohesion scale and institutional assets index scores are above average, and the probabilities for intervention peer interaction, HIV support group attendance, and sex worker group participation are greater than 0.50. Similar to the transgender sample, these assets come from internal and external sources and across the interpersonal, group, and institutional levels. Details for all items across classes are presented in **Table 2**.

Being in the Internal and External Multilevel Assets class was significantly associated with currently taking ART (aOR = 13.10; 95%CI 1.28, 134.26; p<0.05) and marginally associated with ART adherence (aOR = 2.68; 95%CI 0.86, 8.35; p<0.10) and viral suppression (aOR = 2.66; 95%CI 0.83, 8.53; p<0.10) compared to the Low Reported Assets class.

Being in the External Institutional Assets class was significantly associated with currently taking ART (aOR = 7.04; 95%CI 1.36, 36.36; p<0.05) compared to the Low Reported Assets class. It was marginally associated with reduced odds of illicit drug use in the past six months (aOR = 0.36; 95% CI 0.11, 1.20; p<0.10) and increased odds of ART disruption in the past six months (aOR = 3.54; 95%CI 0.93, 13.44; p<0.10). Full details of the associations between class membership and syndemic health outcomes are presented in **Table 5**.

**Table 5:** Distribution of syndemic health outcomes across classes (n(%)) and association between class membership and syndemic health outcomes for cisgender sample with low reported assets class as reference.

	Low Reported Assets	External Institutional Assets	Internal & External Multilevel Assets	Comparison Class	Adjusted^a^ OR (95% CI)	p-value
Proportion of assigned participants	19 (9.00%)	115 (54.50%)	77 (36.49%)			
Viral suppression						
Yes	12 (63.16%)	82 (71.30%)	66 (85.71%)	External Institutional Assets	1.12 (0.39, 3.20)	0.832
No	7 (36.84%)	33 (28.70%)	11 (14.29%)	Internal & External Multilevel Assets	2.66 (0.83, 8.52)	0.099*
ART Adherence in past 4 days						
Perfect adherence	5 (26.32%)	40 (34.78%)	39 (50.65%)	External Institutional Assets	1.40 (0.46, 4.25)	0.553
Not perfect adherence	14 (73.68%)	75 (65.22%)	38 (4935%)	Internal & External Multilevel Assets	2.68 (0.86, 8.35)	0.089*
Stopped taking ART in past 6 months						
Ever	3 (15.79%)	37 (32.17%)	14 (18.18%)	External Institutional Assets	3.54 (0.93, 13.44)	0.063*
Never	16 (84.21%)	78 (67.83%)	63 (81.82%)	Internal & External Multilevel Assets	1.68 (0.41, 6.84)	0.466
Currently taking ART						
Yes	15 (78.95%)	112 (97.39%)	76 (98.70%)	External Institutional Assets	7.04 (1.36, 36.36)	0.020**
No	4 (21.05%)	3 (2.61%)	1 (1.30%)	Internal & External Multilevel Assets	13.10 (1.28, 134.26)	0.030**
HADS-A						
Borderline Abnormal or Abnormal	8 (42.11%)	53 (46.09%)	24 (31.17%)	External Institutional Assets	1.41 (0.51, 3.87)	0.506
Normal	11 (57.89%)	62 (53.91%)	53 (68.83%)	Internal & External Multilevel Assets	0.75 (0.26, 2.17)	0.600
PHQ-9						
Moderate to Severe depression	6 (31.58%)	32 (27.83%)	16 (20.78%)	External Institutional Assets	1.02 (0.35, 2.99)	0.974
Minimal to Mild depression	13 (68.42%)	83 (72.17%)	61 (79.22%)	Internal & External Multilevel Assets	0.71 (0.22, 2.22)	0.551
Physical and/or sexual violence in past 6 months						
Any	2 (10.53%)	15 (13.04%)	7 (9.10%)	External Institutional Assets	1.63 (0.33, 8.03)	0.551
None	17 (89.47%)	100 (86.96%)	70 (90.90%)	Internal & External Multilevel Assets	1.12 (0.20, 6.12)	0.898
Alcohol Use Disorder (AUD) Risk						
At risk	13 (68.42%)	70 (60.87%)	57 (74.03%)	External Institutional Assets	1.06 (0.36, 3.12)	0.922
Not at risk	6 (31.58%)	45 (39.13%)	20 (25.97%)	Internal & External Multilevel Assets	2.06 (0.65, 6.50)	0.218
Illicit drug use in past 6 months						
Any	5 (26.32%)	13 (11.30%)	9 (11.69%)	External Institutional Assets	0.36 (0.11, 1.20)	0.095*
None	14 (73.68%)	102 (88.70%)	68 (88.31%)	Internal & External Multilevel Assets	0.37 (0.10, 1.33)	0.127

* = p<0.10

** = p<0.05

*** = p<0.01.

a = Adjusted for age.

When considering the potential role of syndemics and the ways these health outcomes are connected, we see that cisgender FSWs in the External Institutional Assets class have slightly higher proportions of participants scoring abnormal or borderline abnormal anxiety on the HADS-A and reporting physical and/or sexual violence compared to the Low Reported Assets class (46.09% versus 42.11%, and 13.04% versus 10.53%, respectively). Therefore, we conducted a post-hoc multivariate multinomial logistic regression to further examine the association between membership in the External Institutional Assets class and ART disruption with age, anxiety, and physical and/or sexual violence included as covariates. The odds of ART disruption remained marginally significant (aOR = 3.52; 95%CI 0.93, 13.32; p<0.10); neither of the other syndemic health outcomes included as covariates were significant.

## Discussion

This study highlights the critical role assets play in promoting health, particularly related to HIV continuum of care, among FSWs living with HIV in the DR. While typologies of assets for the cisgender and transgender samples were similar, key distinctions raise questions about the role of gender identity in asset access and ownership.

The most protective pattern of assets for both groups draws from the RAF’s interpersonal, community, and institutional realms and reflects bonding and bridging social capital. When looking at the impact of class membership on syndemic health outcomes, the only consistent results found across samples relate to HIV care and treatment, particularly currently taking ART and ART adherence. This mirrors existing research that shows interventions that include peer engagement, particularly around health care service access and utilization, and community groups or centers for interaction can improve HIV outcomes [30,32–34]. Around the world, community-centered interventions and organic community strategies that address the syndemics FSWs face highlight how assets, particularly social assets, are critical building blocks for harm reduction and promoting health and safety. In particular, evaluations of interventions designed to increase social assets (peer connections and community networks) consistently report improvements in HIV treatment outcomes.

In Tanzania, the Project Shikamana model included peer education, service navigation, and support as well as establishing a community-led drop-in center to facilitate community mobilization and regular communication via text messaging [30,46]. Engagement with HIV care, ART use, and ART adherence was significantly higher among Project Shikamana participants living with HIV compared to the control group [30]. The intervention was found to be effective in part because of the social support offered between the cisgender FSW participants and from peer navigators, which offered knowledge, support, and guidance on how to access HIV care and maintain treatment [31].

In India, programming developed by and for sex workers through the country-wide Avahan program, such as the work by Ashodaya Samithi, a sex workers’ organization in Mysore, focused on community mobilization, creating an enabling environment, and increasing access to and use of services, in part through the introduction of pee educators [32,66,67]. Evaluations found that their programming decreased new HIV infections and improved HIV testing, ART use, and treatment retention [32,66,67].

Finally, in the DR, Abriendo Puertas, an intervention designed to address multiple barriers to HIV care and treatment for cisgender FSWs, which included individual counseling sessions, peer service navigation, and community mobilization among other activities [33], increased ART adherence and reported protected sex among participants [33]. A piloted version of the Abriendo Puertas intervention with transgender FSWs also found improved HIV care and treatment outcomes, including current ART use [34].

LCA does not allow for assessing the impact of the individual variables included in the model to determine what is most impactful. However, the cisgender three-class model suggests that while external institutional assets, or material social capital, are helpful, the addition of interpersonal and community assets, or relational social capital, is how the greatest benefit is achieved. Further, from the transgender two-class model, we can compare external institutional assets alone and when combined with the internal and external multilevel assets class; we again see there are differences in health outcomes and that the addition of interpersonal and community assets improves HIV-related outcomes. Taking these results in combination with the aforementioned research from India, Tanzania, and the DR, there is ample evidence for the need to promote the development of interpersonal and community assets among FSWs as these appear to be the most supportive with regards to HIV care and treatment as well as other health outcomes.

It is also necessary to consider where these interpersonal and community assets are drawn from in order to better understand their impact [16,17,20–22,25]. Given that the focus of this study is on the FSW community, the group membership index, HIV support group attendance, and receipt of cash transfer variables represent bridging social capital as they relate to connections with people outside of the FSW community; intervention peer interaction, social cohesion scale, and sex worker group participation represent bonding social capital as they represent connections among FSWs. Putnam [25] framed bonding social capital as how one gets by while bridging social capital is how one gets ahead. It is possible that we found a significant impact of these patterns of assets on HIV care and treatment outcomes because there are variables representing bridging social capital specifically focused on HIV, enabling participants to get ahead with regard to improving their HIV care and treatment. The HIV support group presents an opportunity for FSWs living with HIV to build their network beyond the sex worker community, which may present relational or material opportunities or benefits that improve their ability to maintain their HIV treatment regimen. When viewed through a narrowly defined lens, the intervention peer engagement variable represents interpersonal support offered to and from a FSW living with HIV, suggesting bonding social capital. But it is possible that this variable, if we expand our view, alludes to bridging social capital as it is a feature of an intervention that brings people from outside of the FSW community together, such as medical providers and advocates, to remove barriers that impede the ability of FSWs living with HIV to receive and maintain care. The improved odds of HIV care and treatment may reflect the presence of bridging social capital that specifically targets HIV outcomes, in part resulting from many participants having previously participated in the Abriendo Puertas intervention [34,46]. Further, it is possible that we did not find similar impacts on the other health outcomes because of the lack of bridging social capital specifically focused on addressing violence, substance use, and/or mental health. While these syndemic health outcomes are related, it might be that interventions focused on one will not inherently or immediately improve the others.

One surprising result from this study was the increased odds of transgender FSWs in their respective most diverse asset class reporting physical and/or sexual violence in the past six months. We hypothesized that the likelihood of holding social and/or economic assets would be protective against violence exposure for FSWs. Past research focused on community-centered responses that enable social connectivity and pooled economic resources suggest more robust and creative responses to violence among FSWs [28,66,67]. For the transgender FSWs who participated in this study, it is possible that the patterns of assets found here might represent bonding social capital related to violence exposure, enabling them to "get by"—i.e. survive or respond to—rather than "get ahead"—i.e. prevent—of violence. While not preventive, the interpersonal and community assets found in the internal and external multilevel assets class may address the stigma associated with violence victimization, which can lead to survivors not reporting [68,69]. If transgender FSWs in the most diverse assets class feel less shame or guilt or receive more social support, they may feel more comfortable reporting these experiences through a survey [70,71]. Another possible explanation is that being more engaged with the sex worker community, particularly participation in a sex worker group, may make individuals more visible within the larger community and thus a target for violence [72]. Future research, particularly using qualitative methods, is needed to investigate this unexpected result and the dynamics between assets and reported violence exposure for transgender FSWs in the DR.

Another surprising result was the increased odds of ART disruption among cisgender FSWs assigned to the External Institutional Assets class compared to the Low Reported Assets class. The post-hoc regression analysis with anxiety and violence included as covariates did not help explain this unexpected result and the potential role of syndemics. Additional research is needed to understand why having external institutional assets would be more harmful than having no assets at all with regards to consistent ART use for cisgender FSWs.

When comparing the two samples, we hypothesized that the cisgender sample would produce a typology of assets that reflect more asset ownership than the transgender sample because cisgender women do not experience the additional burden of transphobia. The opposite was found. The cisgender three-class model showed fewer assets in the additional class. The cisgender patterns of assets might instead reflect the burden of sexism that women assigned female at birth face over the life course that limits their access to and accumulation of assets. The DR ranks 104 in the global Gender Inequality Index (GII) [73], with a score that suggests inequality remains an entrenched problem that disadvantages women and girls [74,75]. While this is not to suggest that transgender women do not face significant discrimination, including sexism, perhaps also for most of their lives if they did not conform to societal expectations for people assigned male at birth, this distinction may partially explain why only the cisgender sample produced the Low Reported Assets class.

Transphobia might also explain why the transgender sample produced two classes that were the opposite of what was hypothesized. Past research shows that transgender FSWs are more isolated, suggesting less bonding social capital within the community [34]. It is possible that the transgender FSWs included in this study are not representative of the larger community given that they were recruited through an earlier pilot intervention, clinic, and peer networks, all of which would at least present the opportunity for participants to have interpersonal and community assets reflecting both bonding and bridging social capital. Other transgender FSWs may have been unable to build the types of social connections that would have increased the likelihood of being recruited because of how transphobia and intersectional stigma related to trans identity isolates and excludes them from society and each other [34], The means of recruitment and resulting sample might explain why the selected model shows that these transgender women are likely to at least hold institutional assets.

Additional research is needed to better understand why some cisgender FSWs are without at least institutional assets, which seem to offer some benefits regarding HIV care and treatment, and the dynamics that inform asset ownership among transgender FSWs, with a particular focus on the role of transphobia.

### Strengths & limitations

This study uses person-centered and asset-based approaches, both of which push the field to consider FSWs and their relationship with the syndemics they face as a result of chronic adversity more holistically. Using LCA enabled us to consider assets, which are connected and reinforcing, as a portfolio rather than as individual items. This has the potential to inform the development of multifaceted, tailored interventions that are better able to meet the needs of FSWs. Furthermore, this study centered FSWs as active, rather than passive, and considered how they are able to individually and collectively respond to the burden of experiencing multiple forms of marginalization. This not only has implications for intervention development that may improve the capacity, connectedness, and overall resilience of FSWs, but shifts away from the deficit-based approach that suggests vulnerable groups can only reduce their risk and not protect themselves or improve their outcomes.

Regarding limitations, this is a cross-sectional study with relatively small non-representative samples. Longitudinal studies are needed in the future to assess the relationships between assets and syndemic health outcomes over time. Further, concerns about power may exist due to the small sample sizes, which might limit the power in detecting the relation between class membership and some syndemic outcomes. However, guidance suggests that more focus should be placed on the specifics of the model and how well the data fits the model than on minimum sample sizes [76]. It is possible that a larger representative sample from multiple sites in the country for both groups may reveal different typologies, including more classes. This could be the result of having more participants and thus greater statistical power, but also because those participants may reflect different experiences than those reflected here.

Regarding measurement, for physical and/or sexual violence victimization, the frequencies for perpetration by regular partners and new and regular clients likely underestimate the true exposure based on a skip pattern in the survey. Only participants who reported having specific partner types in the previous 30 days were asked about physical and sexual violence related to those types of partners in the past six months. Further, two new indices were used to measure certain assets (other community group member and institutional asset ownership), but currently lack validation metrics and require further examination. Finally, this analysis explored a secondary research question from the two companion studies, which were not specifically designed to examine resilience assets among FSWs. It is possible there are other types of interpersonal, community, and institutional assets that FSWs have and use that are missing. More research, particularly utilizing qualitative methods, is warranted to more comprehensively understand the resilience assets transgender and cisgender FSWs in the DR have and how they relate to syndemic health outcomes.

Finally, we employed a syndemic framework to examine a variety of interconnected health outcomes, but we do not conduct a syndemic analysis. Rather than testing disease interaction [77,78], we conducted post-hoc analyses when deemed appropriate to examine how some health outcomes may influence others.

## Conclusion

Cisgender and transgender FSWs living with HIV in the DR have built portfolios of assets that facilitate their resilience in the face of multiple layers of marginalization. This study identified patterns of interpersonal, community, and institutional assets that appear to support FSWs’ ability to maintain their health, including their HIV continuum of care. This work has implications for interventions that seek to expand FSWs’ assets to bolster their ability to care for their and their community’s health and wellbeing. Particular attention should be given to building interpersonal and community assets, both from within and outside of the sex worker community.
